# Supplementary material for: Machine Learning Accelerates Crystallization for Structure Determination
Source: Angew Chem Int Ed Engl. 2026 May 4;65(25):e1218503. doi: 10.1002/anie.1218503 (PMC13266958; doi:10.1002/anie.1218503)

checkCIF/PLATON report

Structure factors have been supplied for datablock(s) lcz069

THIS REPORT IS FOR GUIDANCE ONLY. IF USED AS PART OF A REVIEW PROCEDURE FOR PUBLICATION, IT SHOULD NOT REPLACE THE EXPERTISE OF AN EXPERIENCED CRYSTALLOGRAPHIC REFEREE.

No syntax errors found. CIF dictionary Interpreting this report

Datablock: lcz069

Bond precision:	C-C = 0.0210 A		Wavelength=1.54184
Cell:	a=18.2200(6)	b=16.9582(5)	c=27.3909(7)
	alpha=90	beta=96.637(3)	gamma=90
Temperature:	100 K		

	Calculated	Reported
Volume	8406.5(4)	8406.5(4)
Space group	P 21/n	P 1 21/n 1
Hall group	-P 2yn	-P 2yn
Moiety formula	C70 H42 Ag6 F36 N12 O12 [+ solvent]	2(C15 H3 Ag3 F18 N6), 2(C20 H18 O6)
Sum formula	C70 H42 Ag6 F36 N12 O12 [+ solvent]	C70 H42 Ag6 F36 N12 O12
Mr	2574.38	2574.37
Dx,g cm-3	2.034	2.034
Z	4	4
Mu (mm-1)	12.332	12.332
F000	4992.0	4992.0
F000'	5015.21	
h,k,lmax	22,21,34	22,20,34
Nref	17332	16714
Tmin,Tmax	0.174,0.201	0.513,1.000
Tmin'	0.111	

```
Correction method= # Reported T Limits: Tmin=0.513 Tmax=1.000
AbsCorr = MULTI-SCAN

Data completeness= 0.964            Theta(max)= 74.992

                                                   wR2(reflections)=
R(reflections)= 0.1304( 12137)                     0.3152( 16714)

S = 1.114                  Npar= 1171
```

The following ALERTS were generated. Each ALERT has the format
test-name_ALERT_alert-type_alert-level.
Click on the hyperlinks for more details of the test.

Alert level B

```
PLAT342_ALERT_3_B Low Bond Precision on  C-C Bonds ..............    0.02098 Ang.
```

Author Response: Disordered structure.

```
PLAT780_ALERT_1_B Coordinates do not Form a Properly Connected Set     Please Do !
```

Author Response: The alert is due to a large number of co-crystallized molecules in the which sometimes do not show as a connected set. This is acceptable from a crystallographic point of view.

Alert level C

```
PLAT082_ALERT_2_C High R1 Value .................................       0.13 Report
PLAT084_ALERT_3_C High wR2 Value (i.e. > 0.25) ..................       0.32 Report
PLAT112_ALERT_2_C ADDSYM Detects New (Pseudo) Symm. Elem         A        84 %Fit
PLAT220_ALERT_2_C NonSolvent   Resd 1  F   Ueq(max)/Ueq(min) Range       3.8 Ratio
PLAT362_ALERT_2_C Short  C(sp3)-C(sp2) Bond  C01S     - C02B     .      1.40 Ang.
PLAT362_ALERT_2_C Short  C(sp3)-C(sp2) Bond  C02Y     - C02N     .      1.40 Ang.
PLAT362_ALERT_2_C Short  C(sp3)-C(sp2) Bond  C01I     - C01Q     .      1.41 Ang.
PLAT362_ALERT_2_C Short  C(sp3)-C(sp2) Bond  C01T     - C030     .      1.41 Ang.
PLAT368_ALERT_2_C Short  C(sp2)-C(sp2) Bond  C03H     - C03V     .      1.23 Ang.
PLAT369_ALERT_2_C Long   C(sp2)-C(sp2) Bond  C03S     - C03V     .      1.54 Ang.
PLAT906_ALERT_3_C Large K Value in the Analysis of Variance ......    36.799 Check
PLAT906_ALERT_3_C Large K Value in the Analysis of Variance ......     8.881 Check
PLAT906_ALERT_3_C Large K Value in the Analysis of Variance ......     3.932 Check
PLAT906_ALERT_3_C Large K Value in the Analysis of Variance ......     2.065 Check
PLAT911_ALERT_3_C Missing FCF Refl Between Thmin & STh/L=    0.600        210 Report
                2  0  0,   4 19  0,   7  7  0, -18 10  1,  -7  7  1,  -5 18  1,
               -5 19  1,  -4 19  1,   1 20  1,   2  1  1,   2 20  1,   4 19  1,
                6 14  1,   6 15  1,   7  7  1,  11  6  1,  -5 18  2,  -4 18  2,
               -4 19  2,  -3 19  2,   0  0  2,   0 20  2,   1  1  2,   1 20  2,
                2 20  2,   3 20  2,   5 15  2,   5 19  2,   6 14  2,   6 15  2,
                  (  180 More Missing: see the .ckf listing file)
PLAT971_ALERT_2_C Check Calcd Resid. Dens.  0.94Ang From Ag04           2.21 eA-3
```

```
PLAT971_ALERT_2_C Check Calcd Resid. Dens.  0.92Ang From Ag05          2.19 eA-3
PLAT971_ALERT_2_C Check Calcd Resid. Dens.  1.14Ang From Ag02          2.13 eA-3
PLAT971_ALERT_2_C Check Calcd Resid. Dens.  0.95Ang From Ag01          2.12 eA-3
PLAT971_ALERT_2_C Check Calcd Resid. Dens.  1.06Ang From Ag02          2.08 eA-3
PLAT971_ALERT_2_C Check Calcd Resid. Dens.  0.92Ang From F02J          2.05 eA-3
PLAT971_ALERT_2_C Check Calcd Resid. Dens.  0.89Ang From Ag05          2.05 eA-3
PLAT971_ALERT_2_C Check Calcd Resid. Dens.  1.03Ang From Ag05          2.04 eA-3
PLAT971_ALERT_2_C Check Calcd Resid. Dens.  1.44Ang From F01D          2.02 eA-3
PLAT971_ALERT_2_C Check Calcd Resid. Dens.  1.07Ang From F01Y          2.01 eA-3
PLAT971_ALERT_2_C Check Calcd Resid. Dens.  0.97Ang From Ag03          2.00 eA-3
PLAT971_ALERT_2_C Check Calcd Resid. Dens.  1.08Ang From Ag04          1.94 eA-3
PLAT971_ALERT_2_C Check Calcd Resid. Dens.  1.14Ang From Ag06          1.89 eA-3
PLAT971_ALERT_2_C Check Calcd Resid. Dens.  1.01Ang From F01U          1.82 eA-3
PLAT971_ALERT_2_C Check Calcd Resid. Dens.  0.99Ang From N00W          1.79 eA-3
PLAT971_ALERT_2_C Check Calcd Resid. Dens.  1.30Ang From Ag04          1.77 eA-3
PLAT971_ALERT_2_C Check Calcd Resid. Dens.  0.59Ang From F02L          1.73 eA-3
PLAT971_ALERT_2_C Check Calcd Resid. Dens.  1.23Ang From Ag04          1.72 eA-3
PLAT971_ALERT_2_C Check Calcd Resid. Dens.  1.14Ang From Ag06          1.69 eA-3
PLAT971_ALERT_2_C Check Calcd Resid. Dens.  1.10Ang From N013          1.68 eA-3
PLAT971_ALERT_2_C Check Calcd Resid. Dens.  1.15Ang From F02J          1.63 eA-3
PLAT971_ALERT_2_C Check Calcd Resid. Dens.  1.24Ang From Ag06          1.63 eA-3
PLAT971_ALERT_2_C Check Calcd Resid. Dens.  1.10Ang From C03V          1.61 eA-3
PLAT971_ALERT_2_C Check Calcd Resid. Dens.  1.16Ang From Ag04          1.61 eA-3
PLAT971_ALERT_2_C Check Calcd Resid. Dens.  0.81Ang From C03H          1.61 eA-3
PLAT972_ALERT_2_C Check Calcd Resid. Dens.  0.82Ang From Ag03         -2.28 eA-3
PLAT972_ALERT_2_C Check Calcd Resid. Dens.  0.77Ang From Ag02         -1.91 eA-3
PLAT972_ALERT_2_C Check Calcd Resid. Dens.  1.34Ang From Ag06         -1.77 eA-3
PLAT972_ALERT_2_C Check Calcd Resid. Dens.  0.87Ang From Ag04         -1.76 eA-3
PLAT972_ALERT_2_C Check Calcd Resid. Dens.  0.94Ang From Ag01         -1.75 eA-3
PLAT972_ALERT_2_C Check Calcd Resid. Dens.  0.89Ang From Ag01         -1.71 eA-3
PLAT972_ALERT_2_C Check Calcd Resid. Dens.  0.91Ang From Ag01         -1.67 eA-3
PLAT972_ALERT_2_C Check Calcd Resid. Dens.  0.86Ang From Ag05         -1.66 eA-3
PLAT972_ALERT_2_C Check Calcd Resid. Dens.  0.95Ang From Ag05         -1.66 eA-3
PLAT972_ALERT_2_C Check Calcd Resid. Dens.  0.98Ang From Ag06         -1.64 eA-3
PLAT972_ALERT_2_C Check Calcd Resid. Dens.  0.86Ang From Ag04         -1.64 eA-3
PLAT972_ALERT_2_C Check Calcd Resid. Dens.  1.37Ang From Ag04         -1.63 eA-3
PLAT972_ALERT_2_C Check Calcd Resid. Dens.  0.98Ang From Ag02         -1.60 eA-3
PLAT972_ALERT_2_C Check Calcd Resid. Dens.  0.70Ang From Ag05         -1.60 eA-3
PLAT972_ALERT_2_C Check Calcd Resid. Dens.  0.37Ang From C02P         -1.59 eA-3
PLAT972_ALERT_2_C Check Calcd Resid. Dens.  1.32Ang From N013         -1.59 eA-3
PLAT972_ALERT_2_C Check Calcd Resid. Dens.  1.52Ang From Ag01         -1.58 eA-3
PLAT972_ALERT_2_C Check Calcd Resid. Dens.  0.88Ang From Ag03         -1.55 eA-3
PLAT972_ALERT_2_C Check Calcd Resid. Dens.  1.02Ang From N00H         -1.54 eA-3
PLAT972_ALERT_2_C Check Calcd Resid. Dens.  0.93Ang From Ag02         -1.52 eA-3
PLAT972_ALERT_2_C Check Calcd Resid. Dens.  0.95Ang From Ag04         -1.52 eA-3
PLAT977_ALERT_2_C Check Negative Difference Density on H02H      .    -0.75 eA-3
PLAT977_ALERT_2_C Check Negative Difference Density on H02A      .    -0.34 eA-3
PLAT977_ALERT_2_C Check Negative Difference Density on H02Q      .    -0.47 eA-3
PLAT977_ALERT_2_C Check Negative Difference Density on H02B      .    -0.43 eA-3
PLAT977_ALERT_2_C Check Negative Difference Density on H02C      .    -0.69 eA-3
PLAT977_ALERT_2_C Check Negative Difference Density on H03X      .    -0.32 eA-3
PLAT977_ALERT_2_C Check Negative Difference Density on H03A      .    -0.42 eA-3
PLAT977_ALERT_2_C Check Negative Difference Density on H03C      .    -0.42 eA-3
PLAT977_ALERT_2_C Check Negative Difference Density on H03O      .    -0.63 eA-3
```

Alert level G

```
PLAT002_ALERT_2_G Number of Distance or Angle Restraints on AtSite         21 Note
PLAT003_ALERT_2_G Number of Uiso or U(i,j)  Restrained non-H-Atoms         95 Report
PLAT004_ALERT_5_G Polymeric Structure Found with Maximum Dimension          2 Info
PLAT042_ALERT_1_G Calc. and Reported MoietyFormula Strings  Differ     Please Check
              Calc: C70 H42 Ag6 F36 N12 O12
              Rep.: 2(C15 H3 Ag3 F18 N6), 2(C20 H18 O6)
PLAT083_ALERT_2_G SHELXL Second Parameter in WGHT  Unusually Large     787.05 Why ?
PLAT171_ALERT_4_G The CIF-Embedded .res File Contains EADP Records          2 Report
PLAT172_ALERT_4_G The CIF-Embedded .res File Contains DFIX Records         18 Report
PLAT177_ALERT_4_G The CIF-Embedded .res File Contains DELU Records          4 Report
PLAT178_ALERT_4_G The CIF-Embedded .res File Contains SIMU Records         12 Report
PLAT186_ALERT_4_G The CIF-Embedded .res File Contains ISOR Records          7 Report
PLAT188_ALERT_3_G A Non-default SIMU Restraint Value has been used     0.0100 Report
PLAT188_ALERT_3_G A Non-default SIMU Restraint Value has been used     0.0100 Report
PLAT188_ALERT_3_G A Non-default SIMU Restraint Value has been used     0.0100 Report
PLAT188_ALERT_3_G A Non-default SIMU Restraint Value has been used     0.0100 Report
PLAT188_ALERT_3_G A Non-default SIMU Restraint Value has been used     0.0100 Report
PLAT188_ALERT_3_G A Non-default SIMU Restraint Value has been used     0.0100 Report
PLAT188_ALERT_3_G A Non-default SIMU Restraint Value has been used     0.0100 Report
PLAT188_ALERT_3_G A Non-default SIMU Restraint Value has been used     0.0100 Report
PLAT242_ALERT_2_G Low    'MainMol' Ueq as Compared to Neighbors of       C01I Check
PLAT242_ALERT_2_G Low    'MainMol' Ueq as Compared to Neighbors of       C01Z Check
PLAT242_ALERT_2_G Low    'MainMol' Ueq as Compared to Neighbors of       C029 Check
PLAT242_ALERT_2_G Low    'MainMol' Ueq as Compared to Neighbors of       C02B Check
PLAT242_ALERT_2_G Low    'MainMol' Ueq as Compared to Neighbors of       C02N Check
PLAT242_ALERT_2_G Low    'MainMol' Ueq as Compared to Neighbors of       C02S Check
PLAT242_ALERT_2_G Low    'MainMol' Ueq as Compared to Neighbors of       C02U Check
PLAT242_ALERT_2_G Low    'MainMol' Ueq as Compared to Neighbors of       C030 Check
PLAT242_ALERT_2_G Low    'MainMol' Ueq as Compared to Neighbors of       C033 Check
PLAT242_ALERT_2_G Low    'MainMol' Ueq as Compared to Neighbors of       C039 Check
PLAT242_ALERT_2_G Low    'MainMol' Ueq as Compared to Neighbors of       C111 Check
PLAT299_ALERT_4_G Atom Site Occupancy Constrained at .............        0.5 Check
              F1      F2      F3      F4      F9      F01X    F02C    F037
              F03M    F93
PLAT300_ALERT_4_G Atom Site Occupancy of F01H       Constrained at       0.75 Check
PLAT300_ALERT_4_G Atom Site Occupancy of F25        Constrained at       0.25 Check
PLAT301_ALERT_3_G Main Residue  Disorder ..............(Resd    1)         4% Note
PLAT605_ALERT_4_G Largest Solvent Accessible VOID in the Structure         18 A**3
PLAT720_ALERT_4_G Number of Unusual/Non-Standard Labels ..........        175 Note
              Ag01    Ag02    Ag03    Ag04    Ag05    Ag06    F007    F008
              F009    O00A    O00B    O00C    F00D    F00E    F00F    F00I
              O00J    F00K    F00L    F00M    F00N    O00O    F00Q    O00R
              O00S    N00T    C01E    C023    H023    C02M    N01C    F00U
              F00V    N00W    C02O    C024    H024    C01S    N00G    O00X
              F00Y    O010    O011    N013    C02Y    C034    H034    C02V
              N00P    F015    F016    O017    F019    N01A    N018    C01J
              C02W    H02W    C035    F01B    F01D    F01G    F01H    C01I
              F01K    F01P    C01R    H01R    C01L    C01F    H01F    C01N
              C01W    H01W    C01M    F01U    F01X    F01Y    C01Z    C020
              C027    O028    C029    C02B    F02C    C02D    F02G    C02H
              H02H    F02J    C02K    H02E    H02F    H02G    F02L    C02N
              C02P    C02A    H02A    C01T    N00Z    N00H    C02Q    H02Q
              C01V    C022    H022    C01O    C021    H021    C02F    C02R
              H02B    H02C    H02D    C02S    F02T    C02U    C02X    C02Z
              C03G    H03I    C03U    H03U    C03S    C03X    H03X    C03F
              H03J    C030    C031    H031    C01Q    N014    N012    C025
              C033    C036    H03K    H03M    H03N    F037    C038    C039
```

```
              C03A    H03A    C03C    H03B    H03C    H03D    C03E    C03H
              H03O    C03I    H03P    H03R    H03S    F03M    F03N    C03O
              H03E    H03F    H03G    C03Q    H03Q    C032    H032    C026
              C03B    H03H    C03L    H03L    C03D    C03V    H03V
PLAT802_ALERT_4_G CIF Input Record(s) with more than 80 Characters          1 Info
PLAT860_ALERT_3_G Number of Least-Squares Restraints ............         673 Note
PLAT868_ALERT_4_G ALERTS Due to the Use of _smtbx_masks Suppressed          ! Info
PLAT883_ALERT_1_G Absent Datum for _atom_sites_solution_primary ..     Please Do !
PLAT910_ALERT_3_G Missing FCF Reflection(s) Below Theta(Min)[Deg]=       3.09 Note
               -1  0  1,   0  1  1,
PLAT912_ALERT_4_G Missing # of FCF Reflections Above STh/L=  0.600        377 Note
PLAT913_ALERT_3_G Missing # of Very Strong Reflections in FCF ....          1 Note
                4  0  4,
PLAT933_ALERT_2_G Number of HKL-OMIT Records in Embedded .res File          8 Note
                3  1 30,   3  1 31,   4  1 30,   5  2 29,   6  1 30,  16  1 14,
               20  0  8,  20  1  9,
PLAT941_ALERT_3_G Average HKL Measurement Multiplicity ..........         2.3 Low
PLAT967_ALERT_5_G Note: Two-Theta Cutoff Value in Embedded .res ..      150.0 Degree
PLAT969_ALERT_5_G The 'Henn et al.' R-Factor-gap value ..........      4.464 Note
              Predicted wR2: Based on SigI**2  7.06 or SHELX Weight 28.30
PLAT978_ALERT_2_G Number C-C Bonds with Positive Residual Density.          0 Info
```

```
   0 ALERT level A = Most likely a serious problem - resolve or explain
   2 ALERT level B = A potentially serious problem, consider carefully
  70 ALERT level C = Check. Ensure it is not caused by an omission or oversight
  47 ALERT level G = General information/check it is not something unexpected

   3 ALERT type 1 CIF construction/syntax error, inconsistent or missing data
  80 ALERT type 2 Indicator that the structure model may be wrong or deficient
  20 ALERT type 3 Indicator that the structure quality may be low
  13 ALERT type 4 Improvement, methodology, query or suggestion
   3 ALERT type 5 Informative message, check
```

Validation response form

Please find below a validation response form (VRF) that can be filled in and pasted into your CIF.

```
# start Validation Reply Form
_vrf_PLAT082_lcz069
;
PROBLEM: High R1 Value .................................       0.13 Report
RESPONSE: ...
;
_vrf_PLAT084_lcz069
;
PROBLEM: High wR2 Value (i.e. > 0.25) ..................       0.32 Report
RESPONSE: ...
;
_vrf_PLAT112_lcz069
;
PROBLEM: ADDSYM Detects New (Pseudo) Symm. Elem         A         84 %Fit
RESPONSE: ...
;
_vrf_PLAT220_lcz069
;
```

```
PROBLEM: NonSolvent   Resd 1  F   Ueq(max)/Ueq(min) Range        3.8 Ratio
RESPONSE: ...
;
_vrf_PLAT362_lcz069
;
PROBLEM: Short  C(sp3)-C(sp2) Bond  C01S     - C02B     .       1.40 Ang.
RESPONSE: ...
;
_vrf_PLAT368_lcz069
;
PROBLEM: Short  C(sp2)-C(sp2) Bond  C03H     - C03V     .       1.23 Ang.
RESPONSE: ...
;
_vrf_PLAT369_lcz069
;
PROBLEM: Long   C(sp2)-C(sp2) Bond  C03S     - C03V     .       1.54 Ang.
RESPONSE: ...
;
_vrf_PLAT906_lcz069
;
PROBLEM: Large K Value in the Analysis of Variance ......     36.799 Check
RESPONSE: ...
;
_vrf_PLAT911_lcz069
;
PROBLEM: Missing FCF Refl Between Thmin & STh/L=    0.600        210 Report
RESPONSE: ...
;
_vrf_PLAT971_lcz069
;
PROBLEM: Check Calcd Resid. Dens.  0.94Ang From Ag04            2.21 eA-3
RESPONSE: ...
;
_vrf_PLAT972_lcz069
;
PROBLEM: Check Calcd Resid. Dens.  0.82Ang From Ag03           -2.28 eA-3
RESPONSE: ...
;
_vrf_PLAT977_lcz069
;
PROBLEM: Check Negative Difference Density on H02H      .      -0.75 eA-3
RESPONSE: ...
;
# end Validation Reply Form
```

It is advisable to attempt to resolve as many as possible of the alerts in all categories. Often the minor alerts point to easily fixed oversights, errors and omissions in your CIF or refinement strategy, so attention to these fine details can be worthwhile. It is up to the individual to critically assess their own results and, if necessary, seek expert advice.

PLATON version of 17/09/2025; check.def file version of 17/09/2025

---

duplicate check

No duplication found

Datablock lcz069 - ellipsoid plot

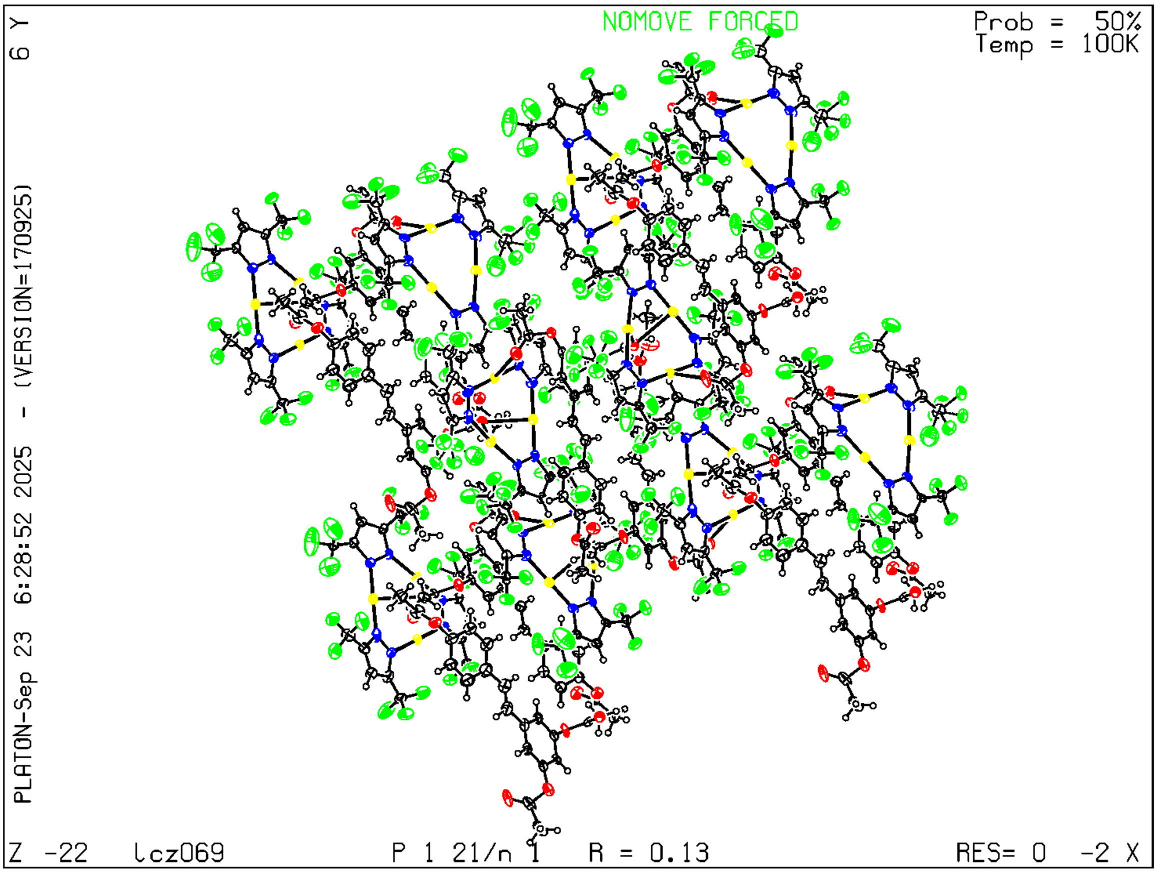

Supplement: Supplementary file 2 — Supporting File 2: anie72462‐sup‐0002‐DataFile.zip. [file ANIE-65-e1218503-s002.zip › supplementary_materials_crystallographic_data submit.zip!/cif_and_checkcif(1-114)/Ag3Pz3_69/Ag3Pz3_69_checkcif.pdf]
